# SOPRA: Scaffolding algorithm for paired reads via statistical optimization

**DOI:** 10.1186/1471-2105-11-345

**Published:** 2010-06-24

**Authors:** Adel Dayarian, Todd P Michael, Anirvan M Sengupta

**Affiliations:** 1 Department of Physics and Astronomy, Rutgers University, Piscataway, New Jersey, USA; 2 Waksman Institute, Rutgers University, Piscataway, New Jersey, USA; 3 BioMaPS Institute for Quantitative Biology, Rutgers University, Piscataway, New Jersey, USA; 4 Current address: Monsanto Company, St Louis, MO USA

## Abstract

**Background:**

High throughput sequencing (HTS) platforms produce gigabases of short read (<100 bp) data per run. While these short reads are adequate for resequencing applications, *de novo *assembly of moderate size genomes from such reads remains a significant challenge. These limitations could be partially overcome by utilizing mate pair technology, which provides pairs of short reads separated by a known distance along the genome.

**Results:**

We have developed SOPRA, a tool designed to exploit the mate pair/paired-end information for assembly of short reads. The main focus of the algorithm is selecting a sufficiently large subset of simultaneously satisfiable mate pair constraints to achieve a balance between the size and the quality of the output scaffolds. Scaffold assembly is presented as an optimization problem for variables associated with vertices and with edges of the contig connectivity graph. Vertices of this graph are individual contigs with edges drawn between contigs connected by mate pairs. Similar graph problems have been invoked in the context of shotgun sequencing and scaffold building for previous generation of sequencing projects. However, given the error-prone nature of HTS data and the fundamental limitations from the shortness of the reads, the ad hoc greedy algorithms used in the earlier studies are likely to lead to poor quality results in the current context. SOPRA circumvents this problem by treating all the constraints on equal footing for solving the optimization problem, the solution itself indicating the problematic constraints (chimeric/repetitive contigs, etc.) to be removed. The process of solving and removing of constraints is iterated till one reaches a core set of consistent constraints. For SOLiD sequencer data, SOPRA uses a dynamic programming approach to robustly translate the color-space assembly to base-space. For assessing the quality of an assembly, we report the no-match/mismatch error rate as well as the rates of various rearrangement errors.

**Conclusions:**

Applying SOPRA to real data from bacterial genomes, we were able to assemble contigs into scaffolds of significant length (N50 up to 200 Kb) with very few errors introduced in the process. In general, the methodology presented here will allow better scaffold assemblies of any type of mate pair sequencing data.

## Background

Next-generation high-throughput sequencing (HTS) holds the promise of revolutionizing the field of biological research [1]. By producing millions of short reads (25-100 bp) per run at a moderate cost, these new sequencing platforms move whole genome sequencing from large centers to individual scientists. To name a few, the list of applications includes gene expression analysis, mutation mapping, non-coding RNA discovery, metagenomics, and protein binding site identification [2,3]. From bioinformatics point of view, there are essentially two types of problems: short read alignment or mapping and *de novo *assembly (the case where no reference genome is available). *De novo *assembly of short reads into larger DNA contigs/scaffolds has proven a bioinformatics challenge both in terms of algorithmic and computational power [4].

Over the past few years, several algorithms have been developed for assembly of short reads. These algorithms can be divided into two broad categories. Some methods, based on 3'kmer extension, use particular data structures to efficiently search for short reads extending a seed sequence [5-7]. In contrast, the graph-based methods pose the sequence assembly as a problem of finding paths on a graph that encodes the short read overlap information [8-11].

Mate pair and paired-end sequencing represent key innovations in short read sequencing that enabled assembly of short contigs into larger scaffolds. Mate pair sequencing was a key innovation that allowed shotgun sequencing of large complex genomes such as humans and Drosophila [12]. Mate pair libraries are generated by enzymatically isolating the ends of a long (1 to 10 kb) DNA molecule. These ends are sequenced in the same direction. In contrast, paired-end sequencing involves sequencing the ends of a smaller (< 600 bp) DNA fragment from both ends in the opposite direction. In this paper, unless we are explicitly contrasting the two methods, we will use the term mate pair to refer to both these technologies.

The current version of some of the above-mentioned short read assemblers can handle mate pair information. However, the use of this information was not central to the concepts that led to the design of most of these algorithms. The sole exception is the ALLPATHS assembler [9], where the use of mate pairs is essential. From a practical point of view, one drawback of ALLPATHS is that it requires at least two paired libraries, with very different insert sizes. Also, the performance of this assembler degrades rapidly as the coefficient of variation of insert size in a library increases past a few percent [9]. This sensitivity is a problem for assembly of real sequence data, as we will see. In the context of previous generations of sequencing technologies with longer reads, the incorporation of mate pair information has also been addressed, either in conjunction with contig assembly [13,14] or as a scaffolding module [15].

Generally speaking, current scaffolding algorithms fall into two categories. Prominent de Bruijn graph based contig building algorithms (e.g. Velvet [8] and Euler [14]) utilize mate pairs to improve the path/walk in the same de Bruijn graph. The other category of scaffolding algorithms [13,15], formulate the problem in terms of graph theoretic constructs in which vertices of the graph are associated to contigs and edges encode mate pair information. Although our approach to the scaffolding problem has partial similarity to this last category, our solution strategy is different, as we will explain. Our algorithm could be implemented, in principle, for any kind of mate pairs, from Sanger reads to the HTS data. However, the special challenges inherent in scaffolding with short read data necessitate an approach that is more sophisticated than those developed so far. That is why we implemented and tested SOPRA in the context of short reads from next-generation technologies.

Existence of repetitive regions in DNA, errors in the sequencing process and mis-assembly of short reads into contigs are all factors which contribute to the complexity of scaffold building using mate pair information. This complexity arises in the form of apparent inconsistency among the set of constraints laid by the mate pairs. Detecting and eliminating the sources of these inconsistencies is essential for the success of any algorithm dealing with mate pair data. This issue is especially important in the context of short read data, since, we expect a higher number of problematic mate pair constraints in the process of scaffold building.

Existing scaffolding algorithms follow a greedy approach, starting with certain schemes of ordering the contigs and pairing information. The mate pairs are then iteratively incorporated as long as the new information does not conflict with the previously assembled scaffolds. In other words, at each step, only a subset of contigs and links in between are considered to improve the assembly. Given the nature of short read data, solution strategies employed in previous studies face difficulties for such data [16].

In this paper, we present SOPRA (Statistical Optimization of Paired Read Assembly), a new tool for de novo assembly of short reads produced by new sequencing platforms. The design of SOPRA is especially targeted to exploit the mate pair information in the process of scaffold assembly. In other words, SOPRA is a module that can be combined with any of the available algorithms for contig assembly. Such a modular design allows greater flexibility and control over the scaffold building process, as has been noted before [15]. SOPRA proceeds in an iterative fashion where at each step problematic mate pair constraints are detected and removed. At each step, one finds a solution consistent with most of the constraints by statistically optimizing over a cost function. Then, one relaxes the most violated constraints. This alternation between removing suspicious data and optimization continues, till we get scaffolds consistent with the remaining trusted constraints.

Among the available *de novo *assemblers, as far as we are aware, Velvet [8] is the only one that can handle color-space data. Adapting available assemblers for color-space data is not a trivial task, since, naive translation from color-space to base-space leads to serious error amplification [17]. Particular attention was paid so that SOPRA could handle data from the SOLiD platform. The final output, given in base-space, is constructed from the color-space assembly, as well as from additional information obtained by translating only the first color call of all the reads. This method will prevent the propagation of the error that can happen in the naive translation. SOPRA is available freely, under the GNU Public License, at http://www.physics.rutgers.edu/~anirvans/SOPRA/

## Results and Discussion

**Figure 1:**
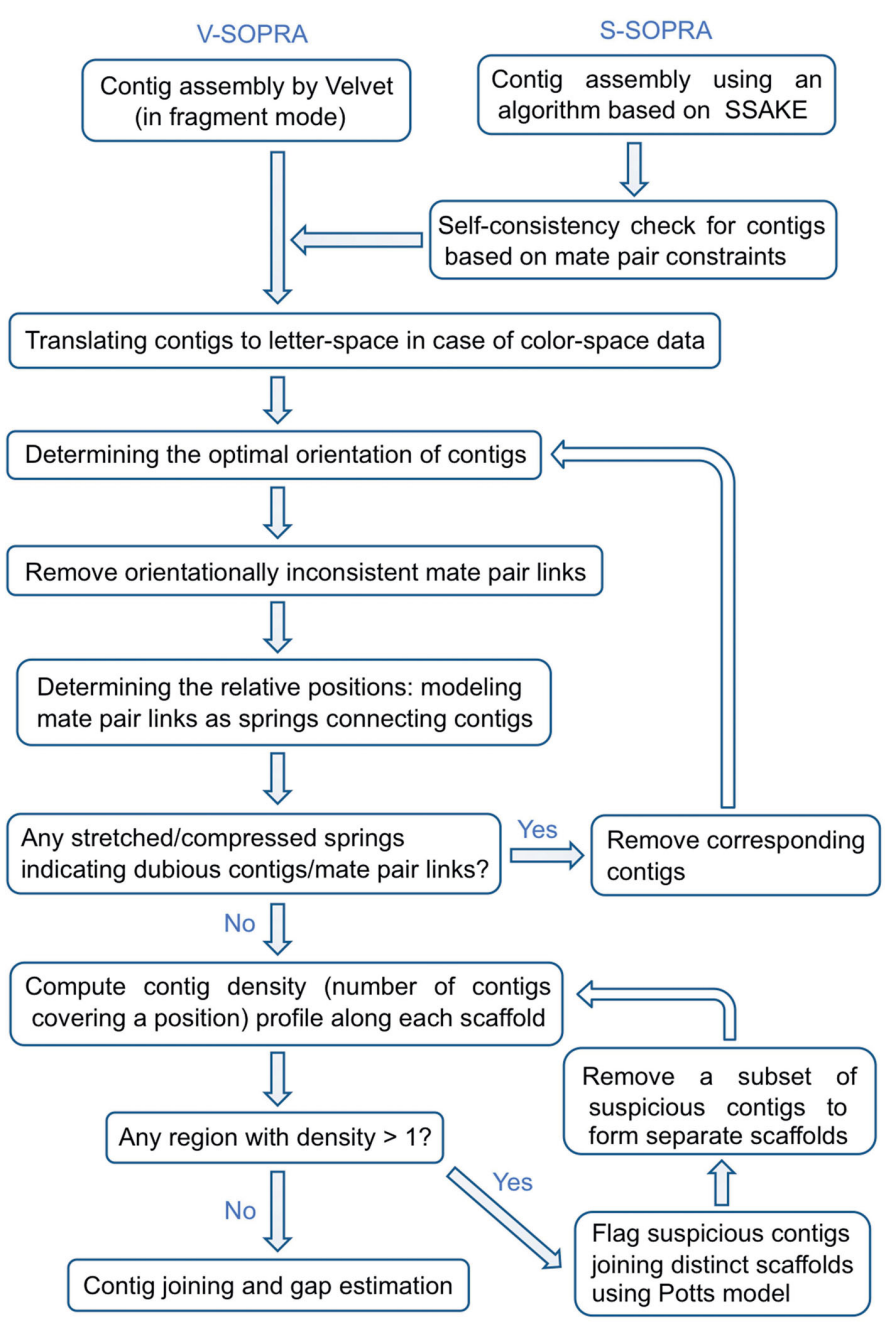
**Flow chart of the algorithm**. In principle, the contig assembly can be performed using any of the available contig assembly algorithms. SOPRA uses the mate pair information to assemble contigs into scaffolds. S-SOPRA and V-SOPRA correspond to the integration of SOPRA with SSAKE and Velvet respectively.

The flow chart of the assembly process is shown in Figure 1. Below, we will explain each section in more details.

## Contig Assembly Preliminaries

As we mentioned, SOPRA is focused on scaffold assembly. The information SOPRA needs from a contig assembler is the computed positions of reads in each contig. SOPRA reconstructs the contigs based on this information. Note that, in the case where these reads do not show perfect overlap, reconstruction of the contigs by SOPRA may not agree with the output of the original contig assembler.

In this paper, we present the performance of SOPRA integrated with two particular contig assembly algorithms, namely, SSAKE [7] and VELVET [8]. We will refer to these two versions as S-SOPRA and V-SOPRA, respectively. This integration is relatively straightforward and described in the Methods section. However, for color-space data, there is one additional step of translating the assembled contigs to base-space.

### Robust translation of contigs assembled in color-space

SOLiD (Sequencing by Oligonucleotide Ligation and Detection) is a novel HTS platform. It uses four fluorescent color probes (coded as 0-3) for reading dinucleotides, namely, two neighboring bases at a time. The sixteen possible dinucleotide combinations are divided into groups of four, each of which is assigned a unique color (e.g. color 2 is assigned to combination AG, GA, TC and CT). However, the groups are designed in such a way that, every combination of the first base and the color call uniquely determines the second base. In other words, each color encodes a transition matrix in the base-space.

Each SOLiD read starts with a reference base, the last base in the primer (usually T or G), followed by a certain number of color calls e.g. G10223...330. Using the reference base and the first color call, we can find the first letter base, which in turn can be combined with the second color call to obtain the second letter base. Continuing so forth, we can translate the whole sequence from color-space to the conventional base-space. The issue is if one of the color calls is wrong (because of an error in the sequencing process), the whole translation from that point on will be wrong. In other words, one error in the color-space will propagate into many errors in base-space. It is because of this error rate magnification that we do not simply translate the SOLiD output directly to the base-space. Instead, SOPRA translates the resulting color-space assembly using a dynamic programming method that avoids such error propagation, as we will explain below.

**Figure 2:**
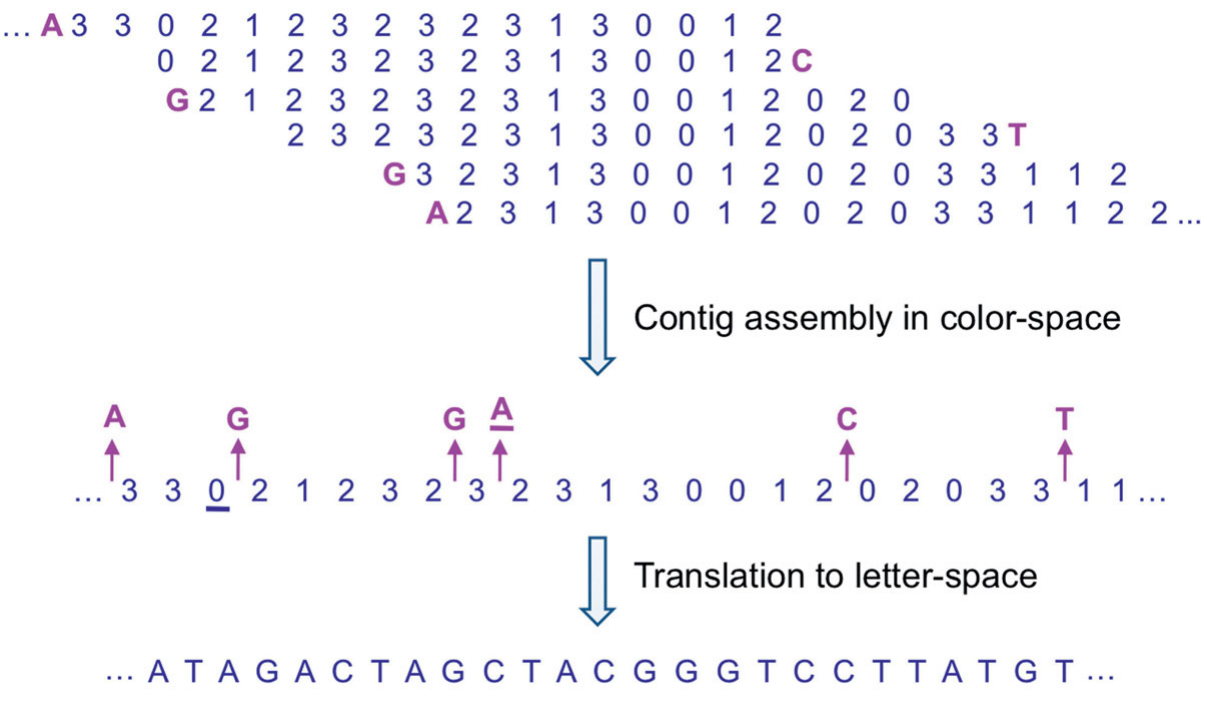
**Robust translation from color-space to base-space**. The base-space suggestions, obtained by translating only the first color call of each read, are shown in magenta. Contig assembly is performed using only the color part (indicated by numbers 0-3) of each sequence. Inconsistencies between the color-space calls and the base-space suggestions, signals the presence of errors. We use an error probability model to find the most likely DNA sequence consistent with this data. The underlined color calls and suggestions in the figure are declared as mistakes in the final translation.

We only translate the first color call (using the reference base) to the base-space but keep the rest of the sequence in color-space. This means a library of sequences, each of which consists of a reference base and *L *color calls, will become a library of sequences that start with a DNA base followed by *L *- 1 color calls. If we ignore for a moment the first DNA base, we can use the *L *- 1 base long sequences for contig assembly in the same way as in regular base-space data. Of course, the result of this assembly will be contigs in the color-space. Although we do not use the first letter base of the sequences in the assembly process, once a sequence is used in building a contig, we record where on the contig the first letter base of the corresponding sequence lies (Figure 2). Notice that the first letter base lies between two color calls and serves as a suggestion for what the DNA base at that position should be. On the other hand, each color call is located between two neighboring DNA bases and provides information about the corresponding dinucleotide.

**Figure 3:**
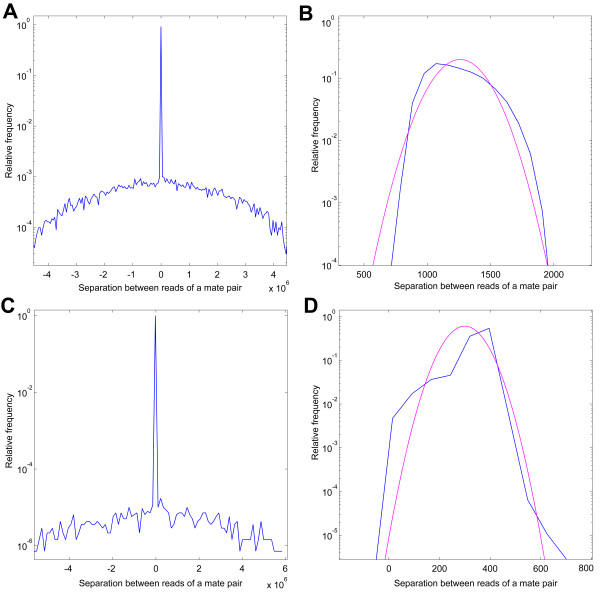
**Histogram of separation between locations of two reads of a mate pair on the reference genome**. This histogram appears to be a combination of two parts. One part is a distribution peaked around the insert size of the mate pair library, as expected. However, in addition, there is a broad background. (A) *E. coli *data from SOLiD platform. (B) *E. coli *dataset, but limited to pairs for which the separation is around the peak region in (A). (C) *P. syringae *data from Illumina platform. (D) *P. syringae *dataset, but limited to pairs for which the separation is around the peak region in (C). Both distributions in (B) and (D) have large standard deviations, each around 20% of the corresponding mean values.

At this point, the assembly result is a sequence in color-space, *C*, plus some letter base suggestions at certain locations of each contig, *F*. In Figure 3, the color-space contig is represented using blue numbers 0-3, whereas, base-space suggestions are shown in magenta. Now, we pose the following question: Given a color-space sequence plus its letter base suggestions, what is the most likely DNA sequence which gave rise to this data? We will set up a model that allows for mistakes in the base suggestions as well as in the assembled color-space contigs. To each arbitrary base-space sequence, the model assigns a probability for that sequence to be the real DNA sequence. The final translation output would be the base-space sequence that maximizes this probability.

The reason why this method prevents propagation of error can be intuitively understood as follows. If the presence of a color call error is ignored, the naïve translation will disagree with most of the base-space suggestions. If this disagreement goes on for a long stretch, from the perspective of the probability function, it is better to declare that particular position to be a color call error and replace it with another color such that the translation becomes consistent with the stretch of base-space suggestions. The ability to alter a color call to enhance the consistency with base suggestions in long stretches helps not only with substitution errors, but also helps to compensate for inconsistency arising from indels. The details of the model are explained in the Methods section.

### Contig self-consistency check

We implemented the self-consistency checks described below only in S-SOPRA. The reason for these checks is that the programs, like SSAKE, which use a greedy algorithm for contig assembly, are particularly vulnerable to generating chimeric contigs. If two legs of a mate pair are located on the same contig, then their relative orientation and position in the contig should match the ones suggested from the mate pair link. If we observe more than certain number of times (threshold is a parameter of the software) cases where the orientation disagrees or the separation between reads is more than one standard deviation different from the insert size, we discard that contig. This method, however, does not necessarily detect chimeric contigs where two or more regions from different parts of the genome have been mis-assembled into one contig. Mate pair information can be used to detect such mis-assemblies, as explained below.

If a contig is genuine, there should be several mate pairs connecting different locations on the same contig (assuming the contig is at least a few times longer than the insert size of mate pairs). However, if it is the case that the contig is composed of two or more sequences coming from different parts of the genome, there should not be as many mate pair links connecting those sequences together. For each point on a contig, we count how many mate pair links connect the right side of that point to the left side. If this number is particularly low for some region, we cut the contig into two at that position.

### Estimation of insert size

In the case where there are enough long contigs, the typical value of the insert size can be estimated from the mate pairs located on the same contig. To do so, we first remove the outliers for which the separation between the pair is different from the suggested insert size by more than the value of the suggested insert size (or equivalently, more than five times the standard deviation, if we assume it is 20% of the suggested insert size). The empirical insert size is equal to the mean value of the separation for the remained pairs. The user needs to know only an approximate value for the insert size based on the library preparation protocol. Prior knowledge of the typical insert size needs to be accurate only when almost all contigs are smaller than the typical inserts.

In case the insert size targeted by the library preparation methods is not available to the user, he/she could take advantage of the empirical distribution of insert sizes output by SOPRA and determine the typical insert size by inspection. In any case, it is a good idea to inspect this distribution, to ascertain the quality of the mate pair library.

### Removal of reads in high coverage regions from scaffolding process

A contig containing repetitive regions can provide conflicting mate pair constraints and cause mis-assembly in the scaffolding process. Although, one could take up the problem of resolving the repeat structures, our approach currently is to identify and remove such contigs from the scaffolding process. One way of detecting repeats is by looking for high coverage regions in each contigs. If a contig has high mean coverage (determined by a parameter of the software) we remove such a contig from scaffold assembly before starting the process. Some contigs have high coverage locally without having high mean coverage. We exclude mate pairs with reads falling in such local high coverage regions for the scaffolding considerations as well (the threshold is a parameter of the software).

## Scaffold Assembly

If two legs of a mate pair are incorporated into two separate contigs, we can infer the relative orientation and relative position of those two contigs on the genome. However, such ordering of contigs is not an easy task, since, the constraints imposed by mate pairs are often not self-consistent. The best one can do is to assign the orientations and positions so that as many constraints as possible are satisfied. In addition, there can be misleading or incorrect information. These dubious constraints arise not only from issues like erroneous contig assembly, but also from innate problems in mate pair data itself.

To elucidate this point, let us examine the two real libraries discussed below in the performance comparison section. In Figure 3, we plot the histogram of separation between the two reads belonging to a mate pair, obtained by matching the reads to the reference genome. As we can see, the distribution of separation could be thought of as a combination of a sharp peak and a broad background that spans over the entire length of the genome. Even if we limit ourselves to the sharp peak (Figures 3B and 3D), the standard deviation is around 20% of the mean value. The variability in separation is much larger than values used for generating simulated data in some studies [8,9]. The algorithm for position assignment has to be robust to such large degree of uncertainties. As will be discussed in the coming sections, in our approach, this goal is achieved by identifying and removing those mate pairs that belong to the broad background as well as from averaging effect of imposing all the remaining constraints together.

For contig building, it is often convenient to represent the sequence overlap information using graph theoretic constructs, e.g. in terms of an overlap graph or a de Bruijn graph. Similarly, it is useful to encode the constraints given by mate pair information into a graphical model. In this model, the underlying undirected graph has vertices corresponding to each contig. Any two contigs connected through mate pairs have an edge in between. We call this graph the contig connectivity graph. This graph is similar to the contig-mate-pair graph introduced in [13], except that here each contig is represented by a single vertex as opposed to two. This kind of graph structure has been used in other studies as well [15]. The structure of the contig connectivity graph, at different stages of the assembly, can be visualized with the help of programs such as GraphViz package [18].

In our formulation, orientations and positions for each contig are variables living on the vertices of this graph. If we introduce the mate pair information as probabilistic constraints on relative orientations and positions of neighboring vertices on the graph, this graphical model has the structure of a Markov random field model [19]. Markov random field models were originally inspired by problems in statistical physics. There are relatively obvious connections between finding the ground state (the most probable configuration of Markov random field) of certain statistical physics models and well-known graph optimization problems as was pointed out by several researchers in the eighties [20]. Such analogies also led to the simulated annealing [21] as a heuristic method for solving hard combinatorial optimization problems (see [22] for a review). We will explain our procedure by invoking the physical analogies, but one could often describe the same procedure using a language familiar to computer scientists.

We perform the scaffolding in two steps. We first assign the orientation of contigs, without considering their positions. Once the orientation is determined, in the second step, we calculate the position of contigs. In this second step, we only use those mate pair links which are consistent with the orientation assigned in the first step. In principle, one could have optimized for orientation and position together, however, our two steps process simplifies the algorithm.

One additional constraint is that distinct contigs cannot be assigned to the same or overlapping positions. This should be true for every possible pair of vertices. This means that if we want to impose this condition in the contig connectivity graph, every possible pair of vertices will be connected by an edge representing this non-overlapping condition. In other words, every vertex will be directly connected to all other vertices. In this sense, the Markov random field structure on the contig connectivity graph is violated. We first solve for orientations and positions ignoring the non-overlapping constraints. The resulting solution typically includes some scaffolds for which the non-overlap condition is not satisfied. We segment these scaffolds into smaller scaffolds satisfying the non-overlap condition using another Markov random field model living on a new graph obtained by augmenting the contig connectivity graph with additional edges between apparently overlapping contigs.

### Determining the relative orientation

We indicate the two possibilities for the orientation of contig *i *by *S*_*i *_= 1 and *S*_*i *_= - 1. If two contigs *i *and *j *are connected through mate pair links, we associate a number to it, denoted by *J*_*ij*_. The sign of *J*_*ij *_is positive if the links suggest that two contigs have the same orientation, otherwise it is negative. The absolute value of *J*_*ij *_is equal to the number of links that connect the two contigs. If all the mate pairs connecting two contigs do not agree with each other, we require that at least a significant majority do. To be a significant majority, we require the percentage of the mate pairs in the dominant group to be higher than a certain threshold, which is a parameter in the software. Otherwise, all the links between those contigs are ignored.

The reason for rejecting all these links is as follow. For two close-by genuine contigs, not belonging to repeats, the source of orientational conflicts is the presence of spurious mate pairs. Usually, these inconsistent spurious links form a small minority. However, when a part of a contig belongs to repetitive regions or one of the contig is chimeric, the nature of the orientational conflicts is different. For example, it is likely that part of the mate pair information suggests the contig belongs to one strand while some other part of the information suggest it belongs to the other strand. In such cases, the majority group and the minority group can have comparable number of links. If a significant majority of links do agree, the minority links are ignored suspecting that they are spurious. If the numbers are comparable, then all links are ignored for the reason mentioned above.

For each configuration of orientations, *S *= (*S*_1_,*S*_2_,...,*S*_*N*_), *N *being the number of contigs, we define the following cost function:(1)

**Figure 4:**
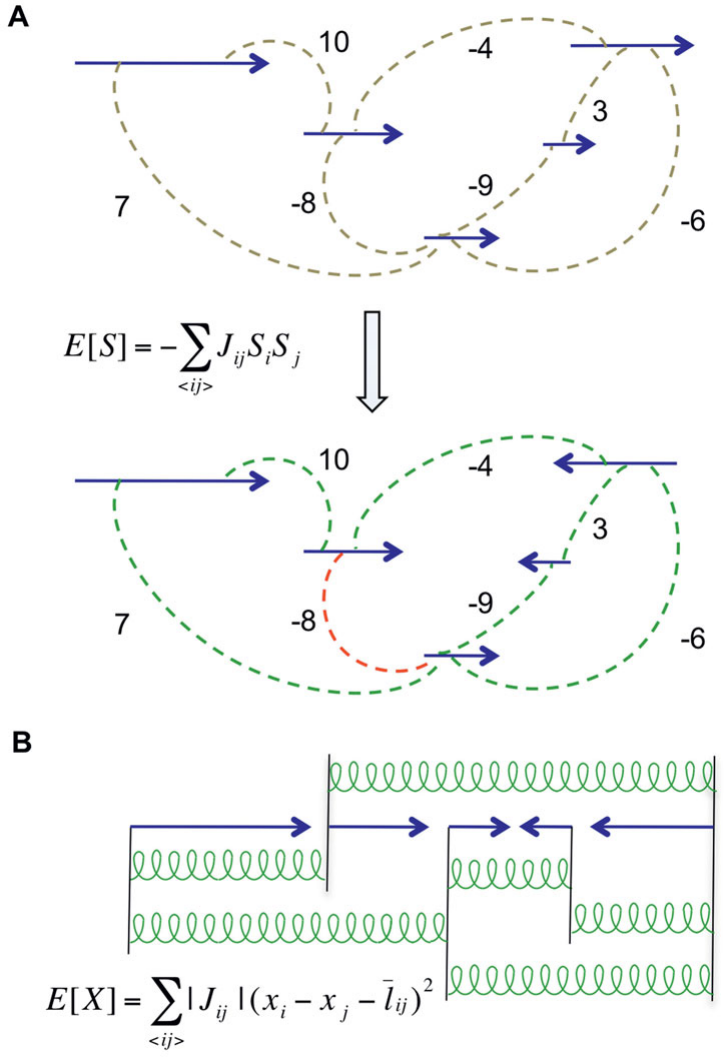
**Modeling constraints on the contig connectivity graph**. (A) For two contigs *i *and *j *connected through mate pairs, the quantity *J*_*ij *_encodes the information about relative orientation (sign of *J*_*ij*_) and number of mate pairs connecting those contigs (absolute value of *J*_*ij*_). Minimizing the energy produces an orientation assignment that satisfies as many constraints as possible. The constraints that are not satisfied in the optimal configuration (shown in red) are ignored in the next part. (B) To determine the relative position of contigs, we model the collection of mate pairs connecting contigs *i *and *j *as a spring attached to the start points of those contigs. The relaxed length of this spring, , is equal to the average suggested distance between the start points of those contigs given by mate pair constraints.

This quantity, a measure of how many of the mate pair links are satisfied, could be thought of as the energy of an Ising spin system with interactions *J*_*ij*_. If it were possible to find a configuration to satisfy all the constraints, we would have: *sign *(*J*_*ij*_) = *sign *(*S*_*i *_*S*_*j*_), ∀ *i*, *j*. The energy of this configuration would be: . As we mentioned before, it is often the case that such a configuration does not exist. Therefore, our goal is to find the best configuration in which as many mate pair links as possible are satisfied. Effectively, we want to find the orientation assignment that minimizes the energy function in Equation (1) (Figure 4A). This minimization is equivalent to the maximum weight cut problem, which appeared in the context of shotgun sequencing [23] and of scaffold assembly [15]. Given that this problem is NP-complete [24,25], it is natural to search for heuristic methods. The approach of these earlier studies is to resolve the constraints in the scaffold assembly problem through particular greedy algorithms that depend upon ad hoc schemes of ordering the contigs. The contrast between such approaches and ours will become clear, as we will explain our algorithm in the Methods section.

### Determining the relative position

For determining the relative positions of contigs, we only use the mate pair links that are orientation-wise consistent with the optimal configuration found in the previous section. Consider a set of contigs connected through mate pair links. Let *X *= (0,*x*_2_,...,*x*_*N*_), denotes the positions of the start points of these contigs. By putting *x*_1 _= 0, we have chosen a particular system of coordinates. Each mate, *r*, connecting contigs *i *and *j*, provides us with some information about *x*_*i *_- *x*_*j*_, encoded in the probability distribution *p*^*r*^(*x*_*i *_- *x*_*j*_). This distribution is picked around certain value, , which can be determined from the location of the two reads in the corresponding contigs and the insert size of the mate pairs (the formula is presented in the Methods section).

Had we not assigned the orientations, one could still define , with the orientations only affecting the sign of the quantity. Note that  is the suggested distance between the corresponding contigs, whereas, the sign determines the ordering (i.e. which one is to the left and which one is to the right). In Figure 4A, next to each edge, we just show *J*_*ij*_'s. However, each edge also carries the additional information on the relative position of the corresponding contigs ('s). Before assigning the orientation, the contig connectivity graph does not fully capture the ordering of contigs, since, as we explained,  is determined up to a sign. After the orientation assignment, the full information about relative position of contigs is captured by this graph.

The overall information provided by all the mate pairs linking contigs *i *and *j *is given by . Note that | *J*_*ij *_| is the number of mate pairs bridging between contigs *i *and *j*. We do not know the exact form of *p*^*r *^(*x*_*i *_- *x*_*j*_); however, if we take it to be a Gaussian centered around , we will have:(2)

where *σ *corresponds to the variance in the insert size of mate pairs. Our approach is to determine the relative position of contigs by maximizing the joint probability distribution:(3)

where  is the average suggested distance between the start points of contigs *i *and *j*. Equivalently, one could minimize the function:

This function has an alternative interpretation as the energy of a coupled system. In this analogy, the collection of mate pairs between two contigs *i *and *j *is replaced by a spring connecting the start points of those contigs. The spring constant is equal to | *J*_*ij *_|, and the relaxed length of the spring is given by . In this way, the original system of contigs connected through a network of mate pairs is modeled as a system of objects connected through a network of springs (Figure 4B). The solution maximizing the probability given in Equation (3) corresponds to the equilibrium position (X*) of the objects in the spring system. These positions could be calculated by solving a set of linear equations corresponding to the force on each object being zero.

In the equilibrium position, if the distance between two contigs is equal to the distance suggested by the mate pairs connecting them, then the corresponding spring is relaxed; otherwise, the spring is either stretched or compressed. In other words, we could define  as a measure of the degree to which the mate pair constraints are violated. If all the suggested distances were self-consistent, all Δ_*ij*_'s would be nearly zero (no stretch/compression in the springs). In real data, it is possible that some sequences match in several locations on the genome, and therefore, mate pair information would not uniquely determine the position of contigs. In our model, the sign of this non-uniqueness is that in the equilibrium solution, X*, some of the springs will be stretched or compressed. The same situation can arise because of contig mis-assembly where two separate regions of the genome are joined into one contig.

When there is a stretched or compressed spring, we remove the contigs attached to the end of that spring from the system and restart the scaffold assembly on the remaining contigs. In other words, we go back to the orientation assignment step (Figure 1). The cycle stops when in the equilibrium position, all the springs are close to their relaxed state, namely, all Δ_*ij*_'s are below a certain threshold. Note that X* is the positions of the start points of contig. If the orientation of contig *i *is positive, it means that it covers the interval  on the scaffold. If *i *has negative orientation, we assign the reverse complement of *i *to the interval .

The greedy algorithms, previously applied to the combinatorially difficult problem of assigning relative positions, consider contigs in a certain order; an order that depends on the number of links associated with each contig [13,15]. Potentially, such methods could be prone to incorporating repeats/chimeric contigs which could have significant number of links associated with it. In contrast, our method has the advantage of providing an unambiguous means for flagging misleading distance constraints with having to commit to any such order.

### Detecting tangled scaffolds by the contig density profile

We calculated the position of the contigs in a scaffold from a set of linear equations based on the assumption in Equation (2). Of course, position intervals corresponding to distinct contigs should be non-overlapping. However, the solution of these linear equations is not guaranteed to satisfy this non-overlap condition. In fact, such overlapping configurations do arise in practice. Below, we explain some of the causes leading to this problem.

**Figure 5:**
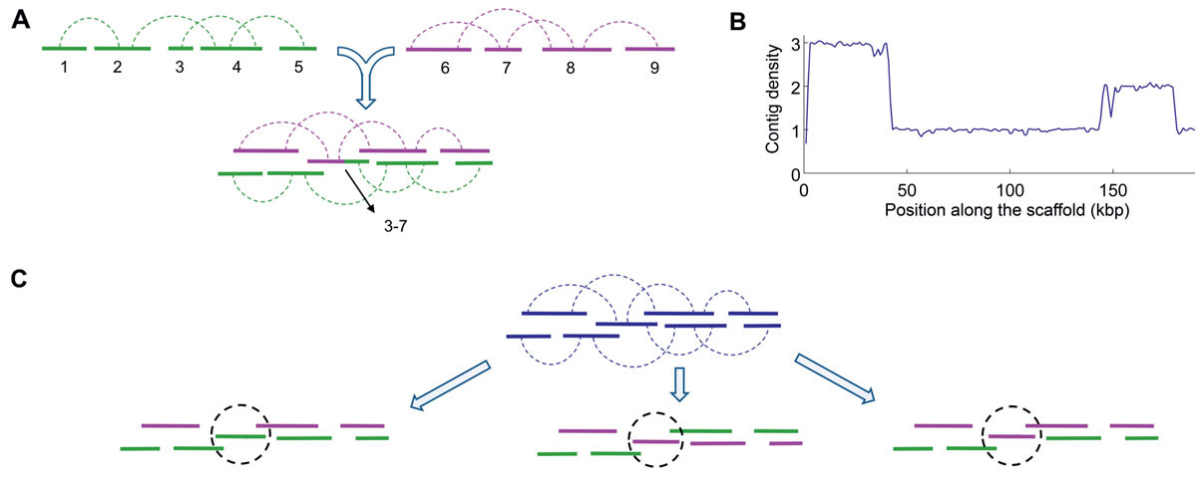
**Detecting and resolving scaffold mis-assembly using density profile and Potts model**. (A) Two scaffolds, shown in green and magenta, belong to the different regions of the genome. Mis-assembly of a chimeric contig composed of contig 3 from the green scaffold and contig 7 from the magenta scaffold causes the two distinct scaffolds to join together. In the new scaffold, many positions are covered by two contigs. (B) For a genuine scaffold, the density profile (see text for definition) should be close to one (or zero for gaps). The plot shows the density profile for a mis-assembled scaffold obtained in the assembly process of a real dataset from the *E. coli *genome. Each point along the x-axis represents a window of length 1000 bases along the scaffold. The y-axis shows the average density for positions located within each window. From this profile, we can infer that at least four scaffolds have been mis-assembled together. (C) Our labeling method for dividing contigs into distinct groups for the case shown in (A) can lead to any of the three possibilities shown here. We use color to present different labels. Note that the problematic contig (3-7) always lies at the boundary between different groups.

Consider the scenario described in Figure 5A. There are two sets of contigs, shown in green and magenta, belonging to distinct regions of the genome. Contigs within each set are self-consistently connected through mate pairs. Assume during contig assembly, contig 3 from the first set and contig 7 from the second set get mis-assembled into one contig. In this case, we obtain a scaffold that contains all the contigs and yet, does not have any stretched or compressed spring.

In addition to contig mis-assembly, existence of repetitive regions in the genome is another factor that can cause improper joining of multiple scaffolds. In that case, contigs 3 and 7 in Figure 5A are seen as one contig in the assembly, whereas they are really copies of the same sequence that matches on multiple places on the genome. Each copy can cause the mis-incorporation of a new set of contigs from its neighbors.

In order to detect this type of complication, we define the 'density profile', a quantity that represents how many contigs cover each region of a scaffold. In the final assembly output, this density should be near one for all regions of each scaffold (except for gaps where the density is zero). For a configuration like in Figure 5A most of the points in the problematic region are covered by two contigs, leading to a higher density. Therefore, by inspecting the density profile, we expect to detect these cases where two or more scaffolds are mis-assembled into one scaffold. Figure 5B shows the density profile obtained in the assembly process of a real dataset from *E. coli *genome (discussed below in the performance comparison section). Notice that there are two regions with density above the background density of one, and that those high densities are in fact very close to integers (3 and 2). The nearly integral values indicate how many potentially distinct scaffolds have been joined together.

### Scaffold segmentation

After detecting high-density regions, we need a procedure to identify and remove the problematic contigs that lead to the merger of disjoint scaffolds. We will call these contigs "junctures" for future references. We wish to assign the rest of the contigs into distinct scaffolds in such a way that each scaffold has an acceptable density profile. With that goal in mind, we provide each contig *i *with a variable *σ*_*i*_. One could think of *σ*_*i*_'s as a putative scaffold label. From the density profile, we can determine *q*, the total number of distinct labels (scaffolds) that we need. For example, the profile in Figure 5B implies *q *= 4.

We want to assign the labels according to two criteria. On one hand, we want the contigs that are directly connected by mate pairs to have the same label. On the other hand, we want the contigs that lie over each other to have different labels. To present these criteria mathematically, we define two matrices *D *and *O*. If contigs *i *and *j *are directly connected by mate pairs, the matrix element *D*_*ij *_is one; otherwise, it is zero. The matrix element *O*_*ij *_is a positive number monotonically increasing with the length of the region that contigs *i *and *j *cover simultaneously. We want to find the label assignment that minimizes the following cost function:(4)

Here,  is the Kronecker delta; it is one if *σ*_*i *_and *σ*_*j *_are equal and zero otherwise. This cost function is exactly the energy of a q-state Potts model with both ferromagnetic and antiferromagnetic interactions. We use a simulated annealing method [21] to find a configuration of label assignment that minimizes the above energy (details explained in the Methods section).

In the minimum energy configuration, neighboring contigs belonging to the same scaffold prefer to have the same label while contigs belonging to different scaffolds, juxtaposed in position space, prefer to have different labels. This is a direct consequence of the two criteria with which we began. However, these two criteria cannot be satisfied everywhere at the same time. Around the junctures, namely, contigs joining such juxtaposed scaffolds, the two criteria are at conflict with each other. The result of this conflict is the formation of domain boundaries (change of label) in the neighborhood of the junctures. To get a better sense of this phenomenon, let us revisit the example in Figure 5A. The result of label assignment by our algorithm could give rise to any of the three configurations in Figure 5C (different labels are shown by different colors). Note that the juncture is always located at the boundary where different labels meet.

Motivated by this discussion, we form an initial list of suspected junctures from the contigs located at label boundaries, namely, contigs having at least one neighbor with a different label. This list often has much fewer members than the original set that we started with. Ideally, one would like to consider the result of removing all the different combinations of suspected contigs from the original set to check if it resolves the problems in density profile. An exhaustive search over all combinations becomes possible when the list is small. Otherwise, one has to limit the list to members located at the densest part of the scaffold. If the list is still too large, we have to proceed with a randomly chosen subset.

After removing any subset of these suspected junctures from the original set of contigs, the remaining set of contigs will form one or more connected components. We score each subset by combining two numbers, one penalizing the formation of too many small components and the other penalizing the presence of high-density regions. We choose the best scoring subset to be removed and focus on the resulting connected components.

For each connected component, we check whether the corresponding density profile is free of high-density regions. All connected components with satisfactory density profiles are declared to be new scaffolds. For the rest, we restart the labeling process individually for each component, and continue this process until all the components have satisfactory density profiles. The removed contigs, either in the Potts model or in the spring model, are reported as single contigs at the end of the assembly.

The Potts model based approach is different from the formulation in terms of non-self-overlapping path introduced in Pop *et al. *[15]. The method of arbitrarily picking the longest non-self-overlapping path [15] through the tangle might end up joining two scaffolds wrongly. In our method, we remove the problematic contigs, even if, in some cases, it could lead to some good scaffold breaking up. If there are mate pairs overarching the removed contigs, it is possible for scaffolds to have the correct continuation. This is the case for the example in Figure 5A, since contigs 6 and 8 are connected by a mate pair overarching contig 7.

### Contig joining and gap estimation

In the last stage of scaffold assembly, we decide whether neighboring contigs in a scaffold are to be joined or be separated by a gap. Notice that according to the computed positions, the end of two neighboring contigs might still have a small positional overlap (the density profile is sensitive only to overlaps larger than a few bases); otherwise, they will be separated by a gap. In either the case of positional overlap or the case where the estimated gap is smaller than certain value (e.g. 10 bases), if the ends of neighboring contigs are similar, we join those two contigs. For the rest of the cases, we insert a sequence composed of letter 'N' between the contigs. The length of each sequence is decided by rounding the length of the corresponding gap to the closest multiple of 50. In the special case where there is no sequence similarity, despite the positions indicating a small overlap, we separate the contigs by a 50 base long sequence of 'N'.

## Assembly Performance on Real Data

### Metrics of assembly quality

Before we discuss our results, we need to define how we assess the quality of a *de novo *assembly. The first obvious measure of performance is the typical size of assembled contigs and scaffolds. This quantity is often reported in terms of an N50 value. Roughly speaking, half of the bases are covered by contigs/scaffolds that are longer than the N50 value. However, N50 provides no indication of the accuracy of the assembled contigs/scaffolds. In order to evaluate the quality of the assembly, it is common to study the performance of the algorithm on data from known genomes. While comparing the assembled components to the reference genome, we need to pay attention to different kinds of errors that could arise and define the metrics of performance accordingly.

To define such metrics, let us bear the following question in mind: In order to map a contig to the reference genome, what type of different operations do we need to do? For example, it might be possible for an entire contig to be matched to a continuous part of the genome with a few mismatches and indels. However, it could also be the case that the contig cannot be matched to a continuous region of the genome; instead, different parts of the contig might match to different regions of the reference genome. Of course, for some contigs, one might not find any significant match at all. In addition to errors in the contigs, there would also be errors in the assignment of relative positions and orientations of contigs in a scaffold.

It is common in the sequence assembly literature to single out mismatch rates and combine some of the other kinds of errors in the 'no-match' category. The emphasis of our algorithm is on using the mate pair information for orienting, positioning and joining contigs. Improper execution of these tasks leads to the formation of chimeric contigs, dislocation and inversion of contigs in a scaffold, as well as merger of distinct scaffolds. Metrics for quality assembly corresponding to these categories of errors are essential for fair comparison among different algorithms. In general, for each algorithm, there is a trade-off between building large scaffolds and making small number of mistakes. For example, a cautious algorithm might produce smaller scaffolds rather than keep on joining suspicious fragments together.

Following the spirit of the above discussion, we will define four categories of errors in order to assess the quality of the assembly. We used MegaBLAST [26] with a minimum identity threshold of 92% to align the sequences against the reference genome (Refseq: NC_007005 for *P. syringae *and NC_010473 for *E. coli*). The sum of the length of all the contigs for which no BLAST hit is found, expressed as a percentage of total assembled bases, is reported as the no-match error rate, *ε*_*no_m*_. Each BLAST hit for a contig comes with a number of mismatches and short indels. Mismatch error rate, *ε*_*mis_m*_, reports the total number of mismatches and indels as a percentage of total assembled bases. In addition, if only some parts of a contig do not match to the reference genome, the total length of those parts contributes to mismatch counts as well.

As we discussed above, there are other types of error that lead to large-scale 'rearrangements' of genomic sequence. The use of the term 'rearrangement error' is inspired by the analogy with the process of genome evolution. Just as local errors in assembly have similarity to mutations and indels, the large scale errors in assembly, have their evolutionary analogues: inversion, translocations etc.

These rearrangement errors, measured in the unit of number of events per Mbp of assembly, are divided into the following categories. The error rate *ε*_*ch *_is associated with chimeric mis-assemblies, namely, the cases where two distinct parts of the genome have been joined into one contig. For chimeric contigs, we would like to differentiate between the cases where the real gap between mis-assembled parts is in the order of few hundred bases and the cases where this gap is in the order of, for example, a few megabases. Therefore, overall error rate *ε*_*ch *_is broken down to two parts,  and , accounting for chimeric contigs involving gaps smaller or larger than 500 bases, respectively.

Apart from the issue of chimeric contigs, we also have erroneous assignment of orientations and positions of contigs in a scaffold. Each time the relative orientation of two neighboring contigs on a scaffold disagrees with that in the reference genome, we have an event contributing to the error rate . In addition, for any two consecutive contigs in a scaffold, we have an estimated separation, which decides the number of 'N' bases we insert in between those contigs in the final output. For consecutive contigs with verified relative orientations, we compare the estimated separation with the real separation on the reference genome. The last category of rearrangement error rate, , is associated with the cases where the difference between those values is greater than 500 bases. The two categories of error, presented in this paragraph, keep track of events where two contigs from different strands or from far apart regions have been brought together.

### Description of the libraries

We present the assembly result for two real datasets, one being a mate pair library from SOLiD, while the other is of the paired-end kind from Illumina. In paired-end technology, mainly used by Illumina, two reads in a pair come from the opposite strands. In mate pair technology, both reads in a pair are from the same strand. The insert size is also typically larger for the mate pair libraries, which is beneficial for many applications. At the same time, owing to the particular enzymatic steps required to make the mate pairs, there is a higher rate of production of molecules which do not represent true ends of the large DNA molecule. The sequence information from these molecules has to be properly identified and handled so as not to lead to inconsistent scaffolds.

The first dataset is a 50 bp mate pair dataset, generated by SOLiD platform, for the 4.7 Mb genome of *Escherichia coli DH10B *http://solidsoftwaretools.com/gf/project/ecoli2x50/. After we used an in-house filter [27] to remove polyclonal and error-laden reads, we were left with 7.4 million pairs of 50 bp long sequences. For this mate pair library, we used the insert size of 1350 bp (Figure 3). Assembly of these reads resulted in very poor quality output. Therefore, we decided to trim down the reads to 35 bp, expecting most of the sequencing errors are concentrated towards the end of the reads [27]. Even after filtering and trimming, the remaining reads provided 100× coverage, and produced better assembly than the raw data set (data not shown).

The other dataset contains 3.5 million pairs of 36 bp long reads from the Illumina platform, providing 40× coverage of the 6.09 Mb genome of *Pseudomonas syringae pv. syringae B728a *[28]. For this paired-end library, we used the insert size of 350 bp (Figure 3).

### Performance comparison

We compare the performance of our algorithm to that of Velvet [8]. One reason for selecting Velvet is that several studies found that the performance of Velvet was either better or at least competitive with other available programs [11,28,29]. The other reason is that we wanted to study the platform dependence of the performance of SOPRA. Velvet is the only program among the popular assemblers that handles color-space data. For *P. syringae *dataset from the Illumina platform, the original study [28] from which we obtained the library has compared performance of several assemblers. The authors attempted assembly using EULER-SR [10] and SHARCGS [5], but they ran out of random access memory (32 Gb available). It also turned out that Velvet outperforms SSAKE [7], VCAKE [6] and EDENA [11]. These last two assemblers do not incorporate mate pair information and were run only in unpaired mode. ALLPATHS [9] requires multiple paired libraries with different insert sizes. Given the above issues, we decided to proceed with comparison Velvet.

In many areas, including biological data mining, a common exercise for assessing the performance of a binary classifier is to consider the DET or ROC curve [30,31]. As one reduces the stringency of the classifier, false negative rate decreases at the cost of increasing the false positive rate. DET/ROC curves provide a quantitative representation of this trade-off and are essential for finding optimal operating point that balances the conflicting goals of keeping both of these error rates down. As we mentioned before, in the context of *de novo *assembly, there is a similar trade-off between N50 and the assembly quality [28]. In this analogy, smaller N50 corresponds to having a high false negative rate, while low quality of the assembly plays the role of high false positive rate.

**Figure 6:**
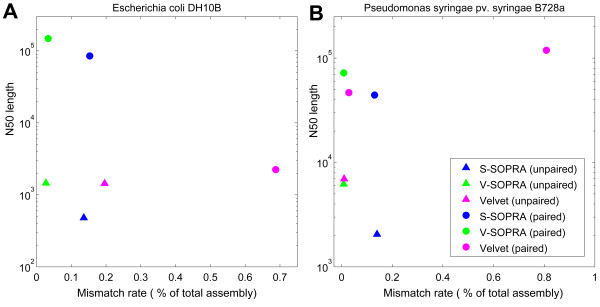
**N50 vs. combined mismatch and no-match error rate for *de novo *assembly of real data**. See main text and the caption for Table 1 for explanation of the error rates.

**Figure 7:**
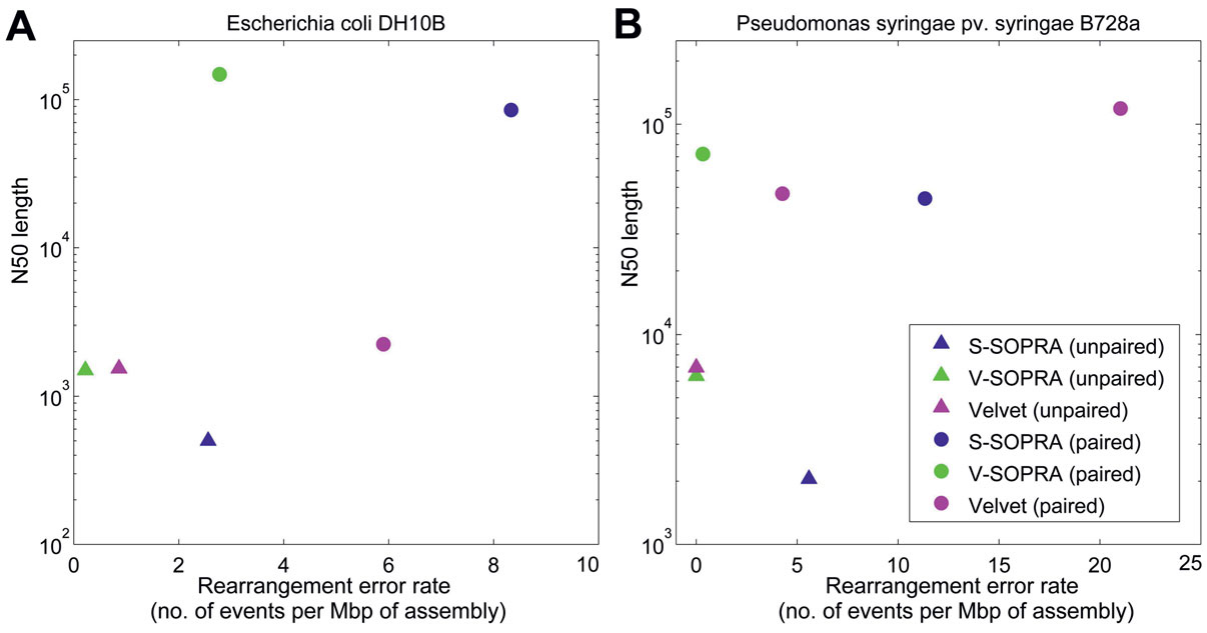
**N50 vs. combined rearrangement error rate for *de novo *assembly of real data**. See main text and the caption for Table 1 for explanation of the error rates

The comparative assembly performance, in the form of N50 versus error rate, is shown in Figures 6 and 7. Ideally, one would like to be on the top left corners of these graphs, which corresponds to large sizes and low error rates. We present the performance of the algorithms both for contig assembly (triangles) and scaffold assembly (circles).

In the case of *E. coli *data produced by SOLiD platform, for contig assembly, the mismatch rate for V-SOPRA is lower than that for Velvet (Figure 6A). This is partly because of error correcting feature of our algorithm for translating color-space data. In contrast, S-SOPRA produces much shorter contigs compared to the other two. Running Velvet with the paired option did not particularly improve the N50, but it increased the mismatch rate significantly. In comparison to Velvet, both V-SOPRA and S-SOPRA perform better in term of scaffold size and error rate, with V-SOPRA outperforming S-SOPRA.

In contrast to the case of the *E. coli *mate pair dataset from SOLiD, pairing information helps Velvet generate much larger scaffolds from the *P. syringae *paired-end Illumina dataset. Figure 6B shows the results of running Velvet, with 'paired' option, on the *P. syringae *reads, for two different parameter sets. Note that the two-fold increase in N50 comes at the cost of increasing the error rate by more than one order of magnitude. This trade-off pattern is consistent with a study comparing, among other things, the performance of Velvet for many combinations of parameters [28]. V-SOPRA produces comparable N50 at a much lower mismatch rate. For this particular dataset, the contig building performance of V-SOPRA and Velvet is nearly identical. Like in the *E. coli *dataset, the performance of S-SOPRA is worse than V-SOPRA.

More or less the same pattern continues with the large-scale rearrangement error rates. In Figure 7 we report N50 versus the combined rearrangement error rates. In the case of Illumina dataset, V-SOPRA did not produce any errors in certain categories (Table 1).

**Table 1:** *De novo *assembly statistics for *P. syringae*

Assembler	*ε*_*no*_*m*_	*ε*_*mis*_*m*_					N50	Genome coverage
	**% of total assembly**		**No. of events/Mbp of assembly**				**Kbp**	**%**

S-SOPRA (unpaired)	.2	.14	.33	5.25	-	-	2.1	98.4
V-SOPRA (unpaired)	.17	.01	0	0	-	-	6.6	97.7
Velvet (unpaired)	.16	.01	0	0	-	-	7	97.2
S-SOPRA (paired)	.3	.13	.49	5.58	0.66	3.12	44.2	98.4
V-SOPRA (paired)	.18	.01	.33	0	0	0	74	97.7
Velvet (paired1)	.16	.02	3.28	.82	0	0.16	46.7	97.7
Velvet (paired2)	.14	.81	4.93	4.1	1.64	7.56	118.8	96.6

The error rate *ε*_*no*_*m *_represents the sum of length of the contigs/scaffolds with no BLAST hit as a percentage of total assembled bases. Mismatch error rate *ε*_*mis*_*m *_reports the total number of mismatches and indels as a percentage of total assembled bases. The error rates  and  are associated with chimeric mis-assemblies, involving gaps smaller or larger than 500 bases, respectively. The error rate  accounts for the number of cases where the relative orientation of two neighboring contigs disagrees with that in the reference genome. The cases where the estimated separation between two consecutive contigs on a scaffold differs from the real separation in the reference genome by more than 500 bases are associated with . These last four categories of errors are measure as the number of erroneous events per megabases of assembly.

In general, for both datasets and all categories of error, our algorithm utilized the mate pair information to enhance N50 by one or two orders of magnitude without significantly increasing the error rates (see details in Tables 1 and 2). The N50 gain from contigs to scaffolds, for the SOLiD dataset is remarkable for SOPRA when compared to the corresponding gain for Velvet. We believe, based on our simulations (data not shown), that our gain for the Illumina dataset would have been much larger if, instead of being around 350 bases, the insert size of this library were close to a kilobase. Another reassuring aspect of SOPRA as compared to Velvet is that for SOLiD dataset, the algorithm managed to keep the mismatch error rate low, partly thanks to the robust handling of the color-space translation.

**Table 2:** *De novo *assembly statistics for *E. coli*

Assembler	*ε*_*no*_*m*_	*ε*_*mis*_*m*_					N50	Genome coverage
	**% of total assembly**		**No. of events/Mbp of assembly**				**Kbp**	**%**

S-SOPRA (unpaired)	.2	.14	.43	2.13	-	-	.5	92.7
V-SOPRA (unpaired)	.02	.03	.22	0	-	-	1.5	94
Velvet (unpaired)	.02	.2	.22	.64	-	-	1.5	94.3
S-SOPRA (paired)	.2	.15	.43	2.13	0.43	2.55	125.5	92.7
V-SOPRA (paired)	.02	.03	.21	0	0.43	1.7	200.6	94
Velvet (paired)	.06	.67	2.55	1.7	0.65	.87	2.3	94.2

For the definition of different error rates, see the caption for Table 1.

We also used MegaBLAST to analyze the contigs which SOPRA removed from the scaffolding process during the assembly. The result is presented in Table 3. For the *P. syringae *dataset from Illumina platform, most of the removed sequences were either chimeric or belonged to repeats (referred to as problematic contigs). For the *E. coli *dataset from SOLiD sequencer, slightly more than half of removed sequences were determined to be problematic. In both cases, the total length of removed sequences remains a small fraction of the total assembly. It should be noted that for a removed contig which was not determined to be problematic, there is a possibility that it contains a short stretch of sequence belonging to repeats which was not identified by MegaBLAST.

**Table 3:** Analysis of contigs removed from the scaffolding process

	*E. coli *dataset		*P. syringae *dataset	
	
	V-SOPRA	S-SOPRA	V-SOPRA	S-SOPRA
Total number of removed contigs	106	338	61	189
Total genomic length of removed contigs (% of total assembly)	192 kb (4.1%)	313 kb (6.7%)	77 kb (1.3%)	272 kb (4.5%)
number of problematic contigs	58	128	60	164
Total genomic length of problematic contigs (% of total assembly)	130 kb (2.8%)	184 kb (3.9%)	76 kb (1.2%)	233 kb (3.8%)

Problematic contigs refer to contigs which are either chimeric, belong to repeats, or do not match to the reference genome. Genomic length means that for repeats, the length is multiplied by the corresponding copy number.

## Conclusion

The goal of scaffold assembly is to arrange contigs such that most of the mate pair constraints are satisfied. Given the inconsistencies in the constraints, any solution strategy inevitably has to decide upon a subset of constraints to be ignored. In our algorithm, this choice is made iteratively, going back and forth between the optimization step and removal of offending constraints. For example, in the process of assigning the optimal orientations, we also detect the links that are not satisfied and are to be removed. The same was true for the next step, where, by modeling the links as springs, we both assign the positions and remove the constraints that cause stretch/compression in this solution.

Taking the entire set of remaining mate pair constraints into account simultaneously at each round of optimization is critical to the success of our approach. Some algorithms, at each step, consider only a small subset of contigs and links in between to improve the assembly in a particular region [13-15]. This manner of local processing of mate pair information stands in stark contrast to our global approach.

In a sequencing project, the issue of large variability in separation of mate pairs (Figures 3B and 3D) has an important implication for the choice of the insert size in the library preparation. The insert size should preferably be large enough to bridge over most of the small repeats or the shallowly sequenced regions. However, as the typical insert size increases, so does the standard deviation of the separation for individual mate pairs. The averaging effect from having multiple mate pairs between two contigs depends upon the number of such pairs, which, in turn, is limited by the size of the corresponding contigs. Therefore, beyond a certain point, larger insert size might result in higher uncertainty in contig positioning. We expect the optimal insert size to be dependent upon the typical size of the contigs, the depth of coverage, and most importantly, the ability to restrict size variation in the library preparation. In our simulations for assembly of some bacterial genomes, the optimal insert size is typically around 1 Kb, if we were to choose only one insert size (data not shown). However, if the contig assembly mostly produces small fragments, namely, the contig N50 is much less than 1 Kb, the quality of scaffold assembly suffers significantly.

In our study, we emphasized the possible conflict between getting larger scaffolds and avoiding mis-assembly. We showed that the N50/error rate trade-off characteristics for V-SOPRA is excellent. In a practical *de novo *assembly project, mis-assembly rates are hard to estimate. As a result, one may be tempted to increase the N50 without consideration of accumulating inaccuracies[32]. Therefore, it is important for such projects to develop a set of independent benchmarks to assess the accuracy of assembly. The N50/error rate trade-off curve, based on such benchmarks, can be used to set the optimal parameters for the assembler.

Currently, SOPRA is quite conservative and it errs on the side of breaking up things whenever there is any confusion. As we have seen, this tendency has not resulted in smaller N50s compared to other algorithms. However, it is possible that a more sophisticated algorithm could partially reconstruct the structure of repeat regions while solving the orientation and positions of different contigs. One may also be able to breakup some chimeric contigs at the right place rather than remove the whole contig. We hope to include these features in the future versions of the algorithm.

The current HTS platforms not only read sequence fragments but also generate additional information regarding relative position and orientation of pairs of reads. Our methodology is particularly adept at exploiting this extra information. The approach developed here could be easily adapted to any new technology that provides additional positional and orientational constraints on multiple reads. Combination of efficient algorithms for utilization of such constraints and improvements in accuracy of reads leading to better quality contig building will bring us closer to the goal of assembling genomes from the next generation of HTS data.

## Methods

SOPRA was implemented in Perl and tested both on a 64-bit Linux and on a Mac OS X server machine. The available memory for both machines was 16 GB. The code is available freely, under the GNU Public License, at http://www.physics.rutgers.edu/~anirvans/SOPRA/

### Robust Translation of Color-space Data

We saw how the output of our color-space contig assembly consists of a sequence in color-space, *C*, plus some base-space suggestions, *F*, at certain locations (Figure 2). However, it may not be possible to find a base-space sequence that agrees with all the color-space calls and base-space suggestions. Therefore, we turn the issue of translating this color-space sequence into a search for the most likely DNA sequence that gave rise to this data (*C *and *F*). Basically, we set up a hidden variable model. The hidden states of the model are the real letter bases. The color calls and letter base suggestions are the observations. There are two unknown parameters: the probability that a given color call is wrong, and the probability that a letter base suggestion is wrong. For the sake of convenience in calculations, we parameterize these two probabilities as  and , respectively.

We can then ask for a given *C*, *F*, *r*_*c *_and *r*_*s*_, what is the probability for a particular base-space sequence, *B*, to be the real DNA sequence? Let *c*_*i *_represent the color call between position *i *and *i *+ 1 of a contig. At each position, we can have different first base suggestions (one for each short read starting at that position). Let *f*_*i,b *_denote the number of times a particular base *b *∈ {*A*, *T*, *C*, *G*} is suggested at position *i*. If at certain position there is no suggestion for a particular base, the corresponding *f*_*i,b *_is equal to zero. Let us represent a base-space sequence of length *N *as *B*_1,*N *_= *b*_1_*b*_2_... *b*_*N*_, where *b*_*i *_∈ {*A*, *T*, *C*, *G*} for all 1 ≤ *i *≤ *N*. For each sequence, *B*_1,*N*_, there is an associated sequence  in color-space such that  is the color associated to the dinucleotide *b*_*i*_*b*_*i*+1_. Let us also represent the probability of *B*_1,*N *_being the real DNA sequence, given *C, F, r*_*c *_and *r*_*s*_, as:

Using the above notation, we have:(5)

 is the Kronecker delta; it is equal to one if the color call between position *i *and *i *+ 1 (i.e. *c*_*i*_) agrees with the color associated with the dinucleotide *b*_*i*_*b*_*i *+ 1 _(i.e. ); otherwise, it is zero. *δ*_*bi*,*b *_is the Kronecker delta as well. The next step is to find the base-space sequence that maximizes the above probability. The particular structure of this model allows us to efficiently solve for the optimal sequence using dynamic programming as follows. Consider an arbitrary position *k*. Equation 5 can be written as:

The middle term on the right hand side contains, which depends on both *b*_*k *_and *b*_*k*__+ 1_. The term *p*_*k *+ 1,*N *_(*B*_*k *+ 1,*N*_) does not contain any variable which corresponds to positions smaller than *k *+ 1, however, it depends on *b*_*k *+ 1_. Similarly, the term *P*_1,*K *_(*B*_1,*K*_) does not contain any variable which corresponds to positions greater than *k*, however, it depends on *b*_*k*_. There are four possibilities for *b*_*k*_, namely, *A*,*T*,*C *and *G*. For each of these possibilities, we can ask what *B*_1,*k-1 *_= *b*_1_*b*_2_...*b*_*k*-1 _will optimize *P*_1,*k *_(*B*_1,*k*_). Imagine we know the answer to this question for some arbitrary *k*. Then, we can easily find the answer to the following question: For each of the four possibilities for *b*_*k*+1_, what *B*_1,*k *_= *b*_1_*b*_2_...*b*_*k *_will optimize *P*_1,*k *+ 1 _(*B*_1,*k *+ 1_)? The reason is that we can write:

For each particular choice of *b*_*k*__+1_, there are four possibilities for *b*_*k*_. For each of these possibilities, we know the first term in the right hand side and we can calculate the second and the third term. The information that we have to save at step *k *+ 1 is that for each *b*_*k *+ 1_, what is the maximum value of *P*_1,*k *+ 1 _(*B*_1,*k *+ 1_) and what base *b*_*k *_corresponds to this value.

We start with *k *= 1 where for each of four possibilities for *b*_1 _we can calculate . We continue as explained above to find, for each of four possibilities for *b*_*N*_, what sequence *B*_1,*N*-1 _= *b*_1_*b*_2_... *b*_*N*-1 _will maximize *P*_1,*N *_(*B*_1,*N*_). We have four options for *b*_*N *_and four corresponding values for *P*_1,*N *_(*B*_1,*N*_). We pick the *b*_*N *_for which the probability *P*_1,*N *_(*B*_1,*N*_) is highest. We then go backward and check, for this choice of *b*_*N*_, what base *b*_*N*-1 _was used. We continue this backward process until we get the whole optimum sequence.

The only remaining issue is the choice of values for *r*_*c *_and *r*_*s*_. Ideally, we would like to choose these values such that the quantity  is maximized. This quantity represents the probability of observing the data, namely, the color-space contig and first base suggestions. One could use iterative methods like expectation maximization in order to find the optimal values of error rates. However, the translation result is robust for a wide range of parameters and training the rate is not particularly essential in all cases that we encountered, for simulated and for real data. Intuitively, the reason for this robustness is as follows. If an error were propagated, it would disagree with most of the subsequent base pair suggestions. The relative strength of *r*_*c *_versus *r*_*s *_decides how many such mismatches would be tolerated before a color call error is declared. If the density of first base suggestion is high, color call errors get found out within a few bases, as long as the ratio *r*_*c *_over *r*_*s *_is within a reasonable range. The density of first base suggestions is usually high for short read data, given the high coverage and the fact that there is one base suggestion for each incorporated short read. As a first estimate, we can put the probability for a letter base suggestion to be wrong equal to, *e*_*s. *_, the sequencing error rate generated by SOLiD platform. The rough estimate for the probability of a color call being wrong would be , where *d *is the average depth of coverage of the corresponding contig.

### Optimization Strategy for Orientation Assignment

We solve the orientation assignment problem by finding the ground state of an Ising model. In general, this is an NP-complete problem [24,25]. However, for moderate quality mate pair data, the typical optimization problems that we face have a redeeming feature. In many cases, most of the vertices in the contig connectivity graph are connected to only a few neighboring contigs, thanks to the nearly linear structure of the scaffold. This feature allows us to partition the graph into smaller components on which the optimization can be performed independently. We can then put the partitioned components back together to find the optimal configuration. Below, we explain this procedure in more detail.

An articulation vertex is defined as a vertex such that by removing it from the graph, the graph splits into two or more disconnected components. For each connected component of the graph, we search for articulation vertices that have more than two neighbors (an articulation vertex with only two neighbors is just part of a linear chain in the graph for which the energy optimization can be solved efficiently). After finding an articulation point, we split the graph into the corresponding disconnected components. We give a copy of the articulation vertex to each of these newly formed components. We iteratively continue this procedure on each of these components until we end up with non-reducible ones i.e. components without articulation points that have more than two neighbors. Finding the articulation points and dividing up the graph takes *O*(*N *^2^) time, where *N *is the total number of the vertices. We can separately optimize the orientation configuration for these non-reducible components. Notice that, in each component, the optimal configuration has a degeneracy of two, namely, if we reverse all the orientations, we get the same energy (*E*[*S*] = *E*[-*S*]).

Once we have the optimized configuration for each of these components, we reverse the process of iterative partitioning. At each step we join back components formed by removal an articulation vertex. Each of these components was provided with a copy of the articulation vertex. Using the freedom of an overall flip within each component, we arrange to have the same orientation for the copies of the articulation vertex in different components. We can stitch the components together by merging the different copies into a single vertex. The order of merging the articulation vertices is the reverse of the order in which they were split. The reason we can find the global optimum solution by separately optimizing non-reducible components and joining them back together is as follows. Given the definition of the articulation points, there is no edge connecting the non-reducible components in the original graph. In other words, in the energy function, there is no term that includes two vertices which belong to different non-reducible components. As a result, the total energy can be broken up into sums of energies of the non-reducible components. Thus, we can optimize the orientational configuration for each of these components separately, up to an overall reversal within each component. The only set of constraints that has to be satisfied is that the copies of each articulation vertex should have the same orientation. This goal can be easily achieved using the freedom of overall reversal within each component.

In order to optimize the non-reducible components, we proceed as follow. For a given component, we pick a random vertex *i *and name the singleton set {*i*} to be *Z*_1_. Next, take all the vertices connected to the vertex in *Z*_1 _and call this new set *Z*_2_. We will then consider all the vertices adjacent to the vertices in *Z*_2_, and for each of them, if it does not already belong to *Z*_1 _or *Z*_2_, we put it in a new set called *Z*_3_. We continue until all the vertices in the corresponding connected component have been visited.

For a general graph, the size of *Z*_*k*_, denoted by | *Z*_*k *_|, grows exponentially as *k *increases. However, for the contig connectivity graph, because of the linear structure of the scaffolds, in many cases | *Z*_*k *_| remains a small number and does not grow as *k *increases. For a given non-reducible component, depending on the sizes of *Z*_*k*_'s, we choose different strategies. In the case where all the sizes are smaller than a threshold value (e.g. six), we use a dynamic programming approach, similar to the Viterbi algorithm, to optimize the energy, *E*[*S*] (Equation 2). In the other case, we use the simulated annealing method as explained below.

The dynamic programming approach is very similar to the procedure explained above for translation of color-space data into base-space. Note that by construction, a vertex belonging to a set *Z*_*k *_can only be connected to the vertices belonging to *Z*_*k*-1_,*Z*_*k *_or *Z*_*k *+ 1_. In other words, we can write:

where the expressions for *E*_1,*k*_,  and *E*_*k+*__1,__*N*_, only contain orientations from vertices belonging to the sets *Z*_1_∪*Z*_2_....∪*Z*_*k*_, *Z*_*k*_∪*Z*_*k*+1 _and *Z*_*k*+1_∪*Z*_*k*+2_...∪*Z*_*N*_, respectively. This means that if we fix orientations of all the vertices belonging to *Z*_*k *_(there are  possibilities for the choice of these orientations), we can optimize *E*_1,*k *_without any knowledge of the orientations associated with vertices belonging to *Z*_*l*_, ∀ *l *>*k*. At this point, it is clear how we can implement the dynamic programming procedure.

Let  be an arbitrary set of orientations for all the vertices belonging to *Z*_*k*_. There are  possibilities for *o*_*k*_. For each of these possibilities, we can ask what choice of *O*_1,*k-1 *_= (*o*_1_,*o*_2_,..., *o*_*k*-1_) will minimize *E*_1,*k*_. If we know the answer to this question for some arbitrary *k*, then, we can easily find the answer to the following question: For each of the  possibilities for *o*_*k*__+1_, what *O*_1,*k *_= (*o*_1_,*o*_2_,...,*o*_*k*_) will minimize *E*_1,*k *+ 1_? The reason is that we can write: . For each particular choice of *o*_*k*__+1_, there are  possibilities for *o*_*k*_. For each of these possibilities, we know the first term in the right hand side and we can calculate the second term. The information that we have to save at step *k *+ 1 is that for each choice of *o*_*k *+ 1_, what is the minimum value of *E*_1,*k *+ 1 _and what choice of *o*_*k *_corresponds to this value.

We start with *k *= 1 where for each of 2 possibilities for *o*_1 _(note that *Z*_1 _only has one member), we can calculate *E*_1,1 _which is equal to zero in both cases. We continue as explained above to find, for each of  possibilities of *o*_*N *_(*N *being the total number of *Z*_*k*_'s), what choice of *O*_1,*N*-1 _= *o*_1_*o*_2_...*o*_*N*-1 _will minimize *E*_1,*N*_. We have  options for *o*_*N *_and  corresponding values for *E*_1,*N*_. We pick the *o*_*N *_for which the energy *E*_1,*N *_is lowest. Note that because of the degeneracy in the energy function (*E*[*S*] = *E*[-*S*]), there are two choices of *o*_*N *_with exactly the same energy. We can arbitrary pick either one of them. We then go backward and check, for this choice of *o*_*N*_, what set of orientation *o*_*N*-1 _was used. We continue this backtracking until we get the optimum orientation for all the vertices.

As mentioned before, for a generic graph, size of *Z*_*k*_'s grow with *k *and the step of going from *k *to *k *+ 1 requires a large number of calculations. This is expected as the problem of minimizing Ising energy on an arbitrary graph is NP-complete [24,25]. However, if the structure of a particular graph allows efficient use of the dynamic programming approach, then the above procedure results in an exact solution. We might have to abandon this method and adopt a heuristic one when there are highly-connected components of moderate or large size.

**Figure 8:**
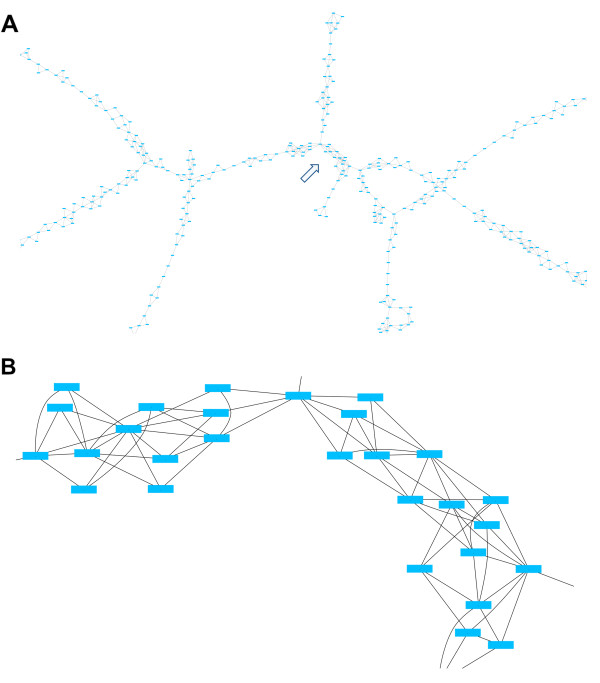
**A typical region of the contig connectivity graph for the *E. coli *dataset**. (A) The graph typically has a sparse structure. Some of the branches shown are part of bigger loops which cannot be seen here. (B) The blow up of the region indicated by arrow in (A).

Figure 8A shows a typical region of the contig connectivity graph for the *E. coli *dataset. As one can see, the contig connectivity graph is mostly quite sparse. Assume if we only consider a small part of the graph, similar to the one shown in Figure 8B, and defines the *Z*_*k *_sets starting from an arbitrary point. Given the typical structure in the graph, it is clear why the size of *Z*_*k*_'s do not often grow as *k *increases. If by removing the articulation points we manage to break up parts of the contig connectivity graph into small components, the above exact method can be applied to most of such components. Some of the branches in figures 8A are part of bigger loops which cannot be seen here. When several such relatively big loops get interconnected, the above optimization strategy often becomes impractical.

### Simulated Annealing Method

We explain the procedure in the context of finding the optimal orientation configuration. Simulated annealing [21] is a Monte Carlo method in which one samples the configuration, *S*, with probability *P*[*S*]∝exp(-*E*[*S*]*/T*), while slowly decreasing the temperature parameter, *T*, towards zero. If the energy of the system reaches a value close to *E*_min _as the temperature goes to zero, it indicates that most of the orientational constraints are satisfied. The advantage of this method over certain greedy approaches is that in simulated annealing, all the contigs and the constraints are treated democratically. Also, in the presence of multiple local optima, one expects simulated annealing to perform better than various domain specific greedy algorithms. In practice, much depends on the particular greedy algorithm and the structure of the graph, as was found in the context of several optimization problems on graphs ([33]). In that study ([33]), it was found that for relatively sparse and regular graphs, simulated annealing did better than some well-established greedy algorithms. This fact, along with many other examples of successful use of simulated annealing [21,22], motivated our choice.

In simulated annealing, we start from an arbitrary configuration, e.g. *S*_*i *_= 1, ∀ *i*. At each step, we randomly choose a contig and check whether by flipping its orientation the energy would decrease or increase. If the energy decreases, we flip the orientation. Otherwise, if the energy increases by Δ *E*, we flip the orientation with probability exp(-Δ *E/T*) where *T *is a parameter. We start with a large value of *T *which will allow orientation flip in most cases. After each step, we slightly decrease *T *according to an exponential cooling schedule [21]. As we go forward, the energy of the system will on average decrease and get closer and closer to *E*_min_. This continues until the energy curve reaches a plateau, at which point the search is stopped.

For the Potts model, the only difference is that, instead of the variable *S*_*i*_, we assign the variable *σ*_*i *_to contig *i*. We start with a random label assignment and at each step we make a decision to whether or not change the label of a randomly chosen contigs to a new randomly chosen label. We find that, although the final label configuration may depend upon the choice of initial configuration, the domain boundaries are robustly reconstructed.

In the optimization problems that we face, if the inconsistencies were too severe, the degree of frustration in the system would be very high, and any heuristic method would typically produce a suboptimal solution. In our experience, this is not the case as evidenced by the fact that the energy of the final orientation configuration is close to the minimum energy (data not shown). This fact, on one hand, allows simulated annealing to find the solution. On the other hand, being able to satisfy most of the constraints indicates that the mate pair data is on the whole trustworthy.

### Calculation of *l*_*ij*_

In a SOLiD mate pair library, each pair is composed of two reads, denotes by *R*3 and *F*3. They come from the same strand and *F*3 read is located to the right of *R*3 as one goes from 5' to 3'. Imagine the *R*3 read was used in contig *i*_*R *_and the *F*3 read was used in contig *i*_*F*_. Now, let us define the variables *τ*_*R *_and *τ*_*F*_. If the *R*3 read itself (and not its reverse compliment) was used in contig *i*_*R*_, then *τ*_*R *_= 1; otherwise, *τ*_*R *_= -1. Similarly, if the *F*3 read itself (and not its reverse compliment) was used in contig *i*_*F*_, then *τ*_*F *_= 1; otherwise, *τ*_*F *_= -1. The position of the *R*3 and *F*3 reads in contigs *i*_*R *_and *i*_*F *_is denoted by *p*_*R *_and *p*_*F*_, respectively. Also, let *Ins *denote the insert size of the library. Then, for the suggested distance between contigs *i*_*R *_and *i*_*F *_(i.e. *x*_*R *_- *x*_*R*_), we have: . Here, . is the orientation assigned to contig *i*_*R*_. For an Illumina paired-end library, the two short reads are located on the opposite strand and face each other. Let us still use the same notation as above, namely, call the first read *R *and the second one *F*, etc. Then, the above formula becomes: . Each mate pair, connecting contigs *i*_*R *_and *i*_*F*_, provides us with its own suggested distance which we calculate using the above formula. The average of all these suggested distances for contigs *i*_*R *_and *i*_*F *_is denoted by .

### V-SOPRA Parameters

For contig assembly part of V-SOPRA, we directly used Velvet v0.7 without invoking the paired option. We get the output in the format of sequence positions in contigs. For base-space data, this information is stored in the *afg *file generated by Velvet. For color-space data, Velvet is part of a pipeline called SOLiD system *de novo *accessory tools [34]. In this pipeline, color-space data has to be preprocessed before inputting to Velvet. Velvet output also has to go through a post-processing step. We use the output of this post-processor that contains the information related to the position of sequences in contigs (the sequences are still in color-space). There is one last step in the pipeline that outputs the final contigs in base-space. However, we do not use this last step. The parameters used for running Velvet in the fragment mode as the first step in V-SOPRA are the same as those described below in the Velvet parameter subsection.

For scaffold assembly, parameter *W *determines the minimum number of mate pairs that have to join two contigs in order for those contigs to be considered connected. For *E. coli *data, we set *W *= 5, whereas for *P. syringae *data we put *W *= 4. Parameter *L*, determining the minimum length that a contig must have in order to be used in the scaffold assembly, was set to *L *= 150 for both datasets.

On the Linux machine, the first step of the program, reconstructing the contigs from Velvet output and recording the mate pair information, took 50 minutes for both *E. coli *and *P. syringae *dataset. The color-space translation for *E. coli *data took 14 minutes. The scaffold assembly part took 1.2 hours for *E. coli *and 5 minutes for *P. syringae *dataset. The runtimes were similar for the Mac OS X server.

### S-SOPRA Parameters

S-SOPRA performs contig assembly based upon our modification of SSAKE v3.2 which can also handle color-space data. The crucial parameter for contig assembly is the parameter that determines the minimum required overlap length between two reads. For *E. coli *data we used *m *= 16, whereas for *P. syringae *data we set *m *= 17. For scaffold assembly, we set *L *= 200 for *E. coli *data, whereas for *P. syringae *data we put *L *= 175. For *E. coli *data, we set *W *= 5, whereas for *P. syringae *data we put *W *= 4.

The first step of the program that builds the contig based on SSAKE algorithm and records the mate pair information took 8.5 hours for *E. coli *and 6 hours for *P. syringae *dataset. The color-space translation for *E. coli *data took 16 minutes. The scaffold assembly part took 7 hours for *E. coli *and 1.8 hours for *P. syringae *dataset. These numbers are for the Linux machine with similar runtime for the Mac OS X server.

### Velvet Parameters

For Velvet, we tried different combinations of parameters and report results for the ones giving the best performance. For *E. coli *data, Velvet in the fragment mode was run with a hash length of 19 and coverage cutoff of 6×. We ran Velvet in the paired mode using a hash length of 19, coverage cutoff of 6× and coverage expectation of 50.

For *P. syringae *data, Velvet in the fragment mode was run with a hash length of 21 and coverage cutoff of 6×. We ran Velvet in the paired mode using two different parameter sets noted by paired1 and paired2 in Table 1 and 2. Both parameter sets used hash length of 21 and coverage cutoff of 6×. The coverage expectation for the first parameter set was 12, whereas for the second parameter set we used 50.

### Filtering Raw SOLiD Data

The performance of any assembler is sensitive to the sequencing error rate. In many cases, for high coverage datasets, assembler performance benefits from filtering the data. The lowered coverage is more than compensated for by the improvement of the data quality. While Illumina data is filtered on the machine, all SOLiD reads are reported. We used an in-house filtering approach for SOLiD data [27] that removed more than 50% of the raw data, still leaving us with 100× coverage.

## Abbreviations

HTS: High throughput sequencing.

## Authors' contributions

AMS and TPM conceived and supervised the project. AD and AMS contributed to the design of the algorithm. AD developed the program and performed the data analysis. All authors read and approved the final manuscript.

## References

[B1] Shendure J Ji H Next-generation DNA sequencing Nat Biotechnol 2008 26 10 1135 1145 10.1038/nbt1486 18846087

[B2] MacLean D Jones JD Studholme DJ Application of 'next-generation' sequencing technologies to microbial genetics Nat Rev Microbiol 2009 7 4 287 296 19287448 10.1038/nrmicro2122

[B3] Mardis ER The impact of next-generation sequencing technology on genetics Trends Genet 2008 24 3 133 141 18262675 10.1016/j.tig.2007.12.007 PMC2680276

[B4] Jones NC Pevzner P An introduction to bioinformatics algorithms 2004 Cambridge, MA: MIT Press

[B5] Dohm JC Lottaz C Borodina T Himmelbauer H SHARCGS, a fast and highly accurate short-read assembly algorithm for de novo genomic sequencing Genome Res 2007 17 11 1697 1706 10.1101/gr.6435207 17908823 PMC2045152

[B6] Jeck WR Reinhardt JA Baltrus DA Hickenbotham MT Magrini V Mardis ER Dangl JL Jones CD Extending assembly of short DNA sequences to handle error Bioinformatics 2007 23 21 2942 2944 10.1093/bioinformatics/btm451 17893086

[B7] Warren RL Sutton GG Jones SJ Holt RA Assembling millions of short DNA sequences using SSAKE Bioinformatics 2007 23 4 500 501 10.1093/bioinformatics/btl629 17158514 PMC7109930

[B8] Zerbino DR Birney E Velvet: algorithms for de novo short read assembly using de Bruijn graphs Genome Res 2008 18 5 821 829 10.1101/gr.074492.107 18349386 PMC2336801

[B9] Butler J MacCallum I Kleber M Shlyakhter IA Belmonte MK Lander ES Nusbaum C Jaffe DB ALLPATHS: de novo assembly of whole-genome shotgun microreads Genome Res 2008 18 5 810 820 10.1101/gr.7337908 18340039 PMC2336810

[B10] Chaisson MJ Pevzner PA Short read fragment assembly of bacterial genomes Genome Res 2008 18 2 324 330 10.1101/gr.7088808 18083777 PMC2203630

[B11] Hernandez D Francois P Farinelli L Osteras M Schrenzel J De novo bacterial genome sequencing: millions of very short reads assembled on a desktop computer Genome Res 2008 18 5 802 809 10.1101/gr.072033.107 18332092 PMC2336802

[B12] Myers EW Sutton GG Delcher AL Dew IM Fasulo DP Flanigan MJ Kravitz SA Mobarry CM Reinert KH Remington KA A whole-genome assembly of Drosophila Science 2000 287 5461 2196 2204 10.1126/science.287.5461.2196 10731133

[B13] Huson DH Reinert K Myers EW The greedy path-merging algorithm for Contig Scaffolding Journal of the Acm 2002 49 5 603 615 10.1145/585265.585267

[B14] Pevzner PA Tang H Fragment assembly with double-barreled data Bioinformatics 2001 17 Suppl 1 S225 233 11473013 10.1093/bioinformatics/17.suppl_1.s225

[B15] Pop M Kosack DS Salzberg SL Hierarchical scaffolding with Bambus Genome Research 2004 14 1 149 159 10.1101/gr.1536204 14707177 PMC314292

[B16] Pop M Genome assembly reborn: recent computational challenges Brief Bioinform 2009 10 4 354 366 10.1093/bib/bbp026 19482960 PMC2691937

[B17] McKernan KJ Peckham HE Costa GL McLaughlin SF Fu Y Tsung EF Clouser CR Duncan C Ichikawa JK Lee CC Sequence and structural variation in a human genome uncovered by short-read, massively parallel ligation sequencing using two-base encoding Genome Res 2009 19 9 1527 1541 10.1101/gr.091868.109 19546169 PMC2752135

[B18] Gansner ER North SC An open graph visualization system and its applications to software engineering Software-Practice & Experience 2000 30 11 1203 1233 10.1002/1097-024X(200009)30:11<1203::AID-SPE338>3.0.CO;2-N

[B19] Kindermann R Snell JL American Mathematical Society Markov random fields and their applications 1980 Providence, R.I.: American Mathematical Society7385929

[B20] Fischer KH Hertz J Spin glasses 1991 Cambridge; New York, NY, USA: Cambridge University Press

[B21] Kirkpatrick S Gelatt CD Jr Vecchi MP Optimization by Simulated Annealing Science 1983 220 4598 671 680 10.1126/science.220.4598.671 17813860

[B22] Laarhoven PJMv Aarts EHL Simulated annealing: theory and applications 1987 Dordrecht; Boston Norwell, MA, USA: D. Reidel; Sold and distributed in the U.S.A. and Canada by Kluwer Academic Publishers3650775

[B23] Kececioglu JD Myers EW Combinatorial Algorithms for DNA-Sequence Assembly Algorithmica 1995 13 1-2 7 51 10.1007/BF01188580

[B24] Garey MR Johnson DS Computers and intractability: a guide to the theory of NP-completeness 1979 San Francisco: W. H. Freeman

[B25] Barahona F On the computational complexity of Ising spin glass models J Phys A 1982 15 3241 3253 10.1088/0305-4470/15/10/028

[B26] Zhang Z Schwartz S Wagner L Miller W A greedy algorithm for aligning DNA sequences J Comput Biol 2000 7 1-2 203 214 10.1089/10665270050081478 10890397

[B27] Sasson A Michael TP Filtering error from SOLiD Output Bioinformatics 2010 26 6 849 850 10.1093/bioinformatics/btq045 20207696

[B28] Farrer RA Kemen E Jones JD Studholme DJ De novo assembly of the Pseudomonas syringae pv. syringae B728a genome using Illumina/Solexa short sequence reads FEMS Microbiol Lett 2009 291 1 103 111 10.1111/j.1574-6968.2008.01441.x 19077061

[B29] Salzberg SL Sommer DD Puiu D Lee VT Gene-boosted assembly of a novel bacterial genome from very short reads PLoS Comput Biol 2008 4 9 e1000186 10.1371/journal.pcbi.1000186 18818729 PMC2529408

[B30] Martin A Doddington G Kamm T Ordowski M Przybocki M The DET Curve in Assessment of Detection Task Performance Proc Eurospeech '97: 1997 1997 1895 1898

[B31] Egan JP Signal detection theory and ROC-analysis 1975 New York: Academic Press

[B32] Salzberg SL Yorke JA Beware of mis-assembled genomes Bioinformatics 2005 21 24 4320 4321 10.1093/bioinformatics/bti769 16332717

[B33] Johnson DS Aragon CR Mcgeoch LA Schevon C Optimization by Simulated Annealing - an Experimental Evaluation. 1. Graph Partitioning Operations Research 1989 37 6 865 892 10.1287/opre.37.6.865

[B34] SOLiD system de novo accessory tools http://solidsoftwaretools.com/gf/project/denovo/

